# A blockchain-based deep learning approach for student course recommendation and secure digital certification

**DOI:** 10.1038/s41598-025-14778-3

**Published:** 2025-08-09

**Authors:** Amjad Rakha, Ahmad Alzubi

**Affiliations:** Institute of Social Sciences, University of Mediterranean Karpasia, Mersin, Turkey

**Keywords:** Intelligent educational system, Students course recommendation, Blockchain-based authenticated certificate system, Deep Certifier-DX509, Computer science, Information technology

## Abstract

Over the past decade, the student course recommendation process with secure certificate issuance has remained a critical research area due to the rise of e-learning and personalized learning. The recommendation system enhances the recommended educational resources to improve the students’ learning process. The previous conventional research works shared hybrid content and collaborative filtering techniques, which boosted academic performance, personalized learning, and secure certification for students. However, the existing techniques faced several difficulties in handling the syllabus updates based on evolving recommendations, complexity, and security issues related to certificate issuance. To address the challenges in the existing techniques, the research introduces the Deep Certifier-DX509 model for secure certificate issuance and student course recommendation. The proposed approach exploits the Modified Attention-Enabled Deep Long Short-Term Memory (MA-DLSTM) Model as a recommendation system to suggest the most suitable courses based on users’ prior academic performance, and integrates X509 as the Certificate generation algorithm. Specifically, the incorporation of the X509 Blockchain with Proof-of-Work (PoW) in the certificate sub-system serves as a major contribution to enhance the security with Two-step authentication and generates accurate course recommendations. Experimental results demonstrate that the proposed Deep Certifier-DX509 model shows superior performance, achieving a high Genuine User Rate (GUR) of 0.73, Memory Usage of 453.81KB, Transaction time of 1.03 s, Responsiveness of 2.39s and Throughput of 119.52bps, outperforming the other existing techniques.

## Introduction

The quick development of the Internet has transformed education and influenced people with online learning. Millions of users register for online courses, specifically open courses such as edX, Coursera, and Udacity^1^. E-learning remains the conventional method, where, with web enhancements, the users achieve the learning objectives. Personalized learning, when compared with e-learning achieved high demand due to easy access to resources at any time and everywhere^2–4^. E-learning and information as well as communication technologies (ICTs) contribute to the SDGs, specifically SDG-4, by promoting virtual or non-face-to-face education^3^. In addition, personalized learning aids tailor individual needs through the abilities and the interests of the users, as the users have massive ideas and expectations^5–8^. The vast amounts of courses accumulated on the Massive Open Online Courses (MOOCs) lead to information overload, specifically in personalized learning, and attract fewer users^9^. Thus, to support easy learning, recommendation systems have been modeled in the past decades that act as a powerful tool to mitigate information overload and provide significant content to the users. The recommendation system utilizes historical data such as views and searches to personalize the learning^9^. Further, to improve the recommendation system and the earning platform, blockchain is used in the research. The blockchain acts as the ledger database that stores the information and encourages encryption to validate the data^10^.

The transformation occurred by converting the outdated conventional CS/IT programs taught at the universities to remote learning, which made a wide open for updating the skills that are highly required for companies to hire executives^11,12^. Due to the unsuitable technologies mounted on the e-learning platforms^13^, there is a need for a large development in the recommendation methodologies that could perform better than the conventional strategies^7^. In conventional research, the performance of the student is assessed with historical data, which is required to be integrated with advanced mechanisms. It has also become a research tool for understanding the mechanisms behind neural networks, similar to attention used in psychology^14^. The recommendation system in^15,16^ provides the most suitable content, which is completely based on the interest of the person, thus minimizing the issue with overloaded information, as most recommendation models utilize vast amounts of data to provide users with significant content^8^. The machine learning (ML) and deep learning (DL) models are introduced in the research area to improve the performance of the recommendation model with an enhanced security level. The drawbacks of ML models are conquered by the DL mechanisms, where the DL models work to improve the efficiency of the research area, and the E-learning Recommendation Architecture (ELRA) bolsters up to prefer appropriate courses based on their individual preferences and business needs. However, the syllabus alterations did not progress in the syllabus and learning materials to enhance the quality of the course.

Further, in cluster-based models, learning resources were not enhanced, and the complexity was not minimized to improve the performance of the model. Ontology-based (OB) content recommender system that could indicate the new user cold-start problem in e-learning systems. The learner’s initial knowledge was gained from the ontology, whereas the initial knowledge represents the level of their knowledge and the style of their learning. However, new technologies were not integrated to analyze the behavior of the learning management system. Personalized Career-path Recommender System (PCRS) that had the capabilities to assist and lead the students who were in high school to select the engineering disciplines. Certainly, the PCRS mimics professional advisors and assists the students by considering their profiles. Furthermore, the PCRS restricts more universities, so the performance of the model was not enhanced. The existing researchers have used many different approaches that struggle to capture the complex relationships between the contexts of the recommendation. Some methods struggle to deal with new users or courses without sufficient historical data. Especially, the conventional approaches employed for course recommendations encountered major drawbacks of long-range temporal dependencies, restricted applications in power-constrained systems, higher computational complexity, challenges in providing personalized recommendations for new users, managing contextual suggestions was computationally demanding for the increased number of users as well as courses, and designing of hybrid architecture required careful tuning and experimentation.

Further, to conquer such drawbacks, the research proposed a Deep Certifier-DX509 to overcome the limitations of the existing methods. The research aims to work with the course recommendation and the blockchain-based certificate issuing process. The Deep Certifier-DX509 is utilized as the proposed model that ensures the authentication of the digital certificates, as well as the course recommendation models. The major contributions of the proposed model are as follows,

### Modified attention-enabled deep long Short-Term memory

The MA-DLSTM is the recommendation model that ensures the recommendation of the most appropriate courses. The research model is the integration of the Deep LSTM along with the modified attention mechanisms, such as spatial and positional attention mechanisms, which can dynamically prioritize the most pertinent spatial and positional features within a user’s past learning activities and course attributes, enabling more precise and personalized course suggestions with the DLSTM.

### Deep certifier-DX509

In the proposed approach, Deep Certifier-DX509 is the model combination of recommendation and the certificate issuance model. The X.509 certificate generation process securely binds a user’s public key to their verified identity within a digital certificate, enabling the validation of authenticity during online communication, which is capable of securing data transfer as well as providing digital identity verification, thus improving the reliability of the model. Further, the incorporation of PoW in the model obtained a highly secure blockchain network.

The research article is comprised of Sections, such as Section “Literature review” is a discussion of the conventional methods, Section “Course recommendation and certificate issue system with deep certifier-dx509” is the description of the proposed Deep certifier-DX509 model with all phases, Section “Results” is a discussion of results, and Section “Conclusion” concludes the research.

## Literature review

Sadia Ali et al.^17^ designed an ELRA framework to prefer the appropriate courses based on their isolated fondness and business urges. The ELRA guided both the learners and tutors to identify the appropriate exclusive course that enhanced their skills and knowledge according to their requirements. Similarly, ELRA diminished the surety and contact between consumers and the education team. However, the alterations were not applied to the syllabus and learning materials to enhance the course.

Sundaresan Bhaskaran et al.^7^ focused on a Cluster-based linear pattern algorithm that involuntarily evaluated the styles of the learner and the learner’s characteristics in certain dimensions. In the model, the learners were well satisfied, and maximum largest clustering sequences were recognized in the cluster-based linear pattern of the mining strategy, respectively. However, learning resources were not enhanced, and the complexity was not minimized to improve the performance of the model.

Joy Jeevamol et al.^13^ drew out an OB content recommender system that represented the new user cold-start problem in e-learning systems. The learner’s initial knowledge was gained from the ontology. However, the method lacked applications of innovative strategies to analyze the behavior of the learning management system.

Manar Qamhieh et al.^18^ demonstrated a PCRS that could assist and lead students who were in high school to select engineering disciplines. The PCRS contained the student’s academic details, personality, and extracurricular activities. Certainly, the PCRS mimicked professional advisors and assisted the students by considering their profiles. Furthermore, the system’s ability to generate tailored career recommendations was hindered by the narrow scope of its data, even after incorporating additional universities and departments, which demonstrated a need for exploring additional strategies to fully optimize its capabilities.

Samina Amin et al.^8^. implemented a Reinforcement Learning (RL) based smart e-learning framework with a Markov Decision Process (MDP) that provided an effective learning path for each student to build up the learning experience. Similarly, the MDP considered the feedback of the learners to identify the new learning paths and the activities to adapt the recommendation method. However, new paths of optimal learning were not framed by considering the sequential behavior of the adaptive learners.

Jingjing Wang et al.^9^ utilized Top-N personalized Recommendation with Graph Neural Network (TP-GNN) in the Massive Open Online Course (MOOCs) that appraised sequential behavior of the user and the general preferences, which hold on the node’s implicit relations and the current preferences of the user. The real-world dataset was utilized to enhance the preference of the model. The model addressed the limitations, such as poor consideration of more learning processes.

Nhi N.Y. Vo et al.^12^. developed a Natural Language Processing (NLP) based course recommendation system that embedded a new method named entity recognition. The model provides satisfaction for the students by enhancing the efficiency of the program by determining the course and job. However, the outcome of the model was not accurate under certain constraints.

Bashir Khan Yousafzai et al.^19^ employed a Hybrid Deep Neural Network (Hybrid DNN) that effectively predicted the performance of the students from the traditional data. The performance of the model was improved by sequentially updating the academics, universities, and departments which was related to the student’s skill. The outcome of the model yielded better efficiency and high scores in the prediction of student results. However, the model was applied only to the single domain, and limited features were considered for a prediction.

Subha S et al.^20^ introduced a hybrid deep learning (HDL) model using a convolutional neural network (CNN), residual network (ResNet), and long short-term memory (LSTM) for the course selection of the candidates in online platforms. The model recommendation system selects the most appropriate course that can encourage students to base their selection on informed decision-making. However, the method required higher computational time and was restricted to a larger database.

Yinping Ma et al.^21^ developed a Deep Personalized Course Recommendation System (DORIS) for course recommendation. It selects the most appropriate courses for students according to their basic information, interests, and the details of all courses. However, the model failed to mitigate the limitations associated with making recommendations for new users or items with minimal interaction data.

Zhou, J., et al.^22^ established a KT_Tranformers model with the combination of the InfoNCE loss framework for course recommendation, which acts as an effective mechanism in generating robust representations of courses and users, accurately reflecting their interconnectedness. Nevertheless, the model encountered a drawback of higher computational cost as well as an overfitting issue, which impacted the effectiveness of the model.

Ren, X., et al.^23^ presented a Long Short-Term Memory with an attention mechanism, which was specifically designed to analyze multimodal data. The course recommendation system effectively mined important temporal and dimensional features to personalize recommendations. Nonetheless, the method was sensitive to parameter tuning, which was not ideal for applications requiring fast processing with large datasets.

Bagunaid, W^24^. , developed an E-FedCloud model, which utilized intelligent algorithms to identify individual student needs and proactively suggested customized learning resources, maximizing performance. Moreover, the method faced complexity in executing multi-modal biometric data, computational complexity, as well as limited application in resource-constrained environments.

Hussain, T^25^. , implemented a hybrid deep-learning model, which revolutionizes e-learning by delivering customized content that aligns with each student’s preferred learning approach, driving greater student success. However, the method had a drawback of potential lack of generalizability to different age groups of learners, particularly within tertiary education, which could restrict the depth of insights gained regarding e-learning influences. The comparison of previous literature works are analyzed in Table 1, given as follows.

**Table 1:** Overall analysis of literature works.

Reference	Technique	Dataset	Advantages	Disadvantages
Sadia Ali et al.^17^	E-learning Recommendation Architecture (ELRA)	Data of teachers and students from the ELRA management system	Reduced the chances of inappropriate course selection	Lack of virtual assistance during course sessions
Sundaresan Bhaskaran et al.^7^	Cluster-based recommender	Sample dataset	Effective in obtaining a better Recommendation for learners.	Complexity in dynamic and hybrid recommendations
Joy Jeevamol V. G and Renumol^13^	CF + CB + ontology model	Real-world dataset	Better performance in cold start conditions generates reliable and personalized recommendations	Course recommendation is not relevant to learners’ behavioral analysis
Manar Qamhieh et al.^18^	Personalized Career-Path Recommender System (PCRS)	PCRS collecting Personalized data of students	Personalized recommendations based on the profile of a student’s characteristics	Difficult recommendation for social-economic factors
Samina Amin et al.^8^	The Massive Open Online Courses (MOOCs)	Real-world data	Interact continually to address learners’ requests, easily interface and interact with other systems.	Complexity and convergence problems by employing conventional RL
Jingjing Wang et al.^9^	TP-GNN (Top-N personalized course recommendation method based on graph convolution network)	Real-world MOOC dataset	Robust to noisy data, the sample mechanism avoids overfitting	Learning progress and learner characteristics affect the model’s performance
Nhi N.Y. Vo et al.^12^	In-domain CSIT-NER model	StackOverflow and GitHub datasets	Strong societal benefit to the higher-tech education sector	Inappropriate recommendation for cross-domain applications
B.K. Yousafzai et al.^19^	Attention-based BiLSTM model	Student Grade Prediction Dataset	Yielded the best results with the baseline work	The dataset is confined to a single domain, and significant features are considered for prediction.
Subha.S et al.^20^	The Hybrid Deep Learning (HDL) model	Real-time Student dataset	An effective solution for solving real-time problems, with better robustness and improved accuracy	Lack of interpretability
Yinping Ma et al. Jilei Zhou et al.^21^	Deep Personalized Course Recommendation System (DORIS)	Anonymized dataset	Make the course’s introduction, prerequisites, and high performance	Cannot solve the cold start problem and overfitting
Jilei Zhou et al.^22^	Time-aware Transformers and knowledge graph	real-time dataset	Increase user engagement and reduce dropout rate.	Higher computational cost and overfitting issues.
Xinwei Ren et al.^23^	LSTM + Attention model	Real online learning MOOC dataset	Effectively reducing the time for users to choose courses and realizing the Personalized Course recommendation service.	Most of the implicit feedback data of users in daily life is difficult to capture
Wala Bagunaid et al.^24^	E-FedCloud system	Open University Learning Analytics Dataset (OULAD)	Ensuring robust authentication, enhancing the quality and responsiveness of e-learning platforms	Dependency on high-quality, multi-modal biometric data raises privacy concerns among users.
Tahir Hussain et al.^25^	Felder–Silverman Learning Style Model (FSLSM)	Publicly available dataset	Effectively uncovers key aspects and sentiments within the feedback, deliver more personalized, responsive, and impactful e-learning experiences	The sentiment analysis approach deals only with English language comments and is less effective with other languages.

### Challenges

- The improper choice of ELRA leads to suboptimal embeddings that struggle to capture the complex relationships between the recommendation contexts^17^.
- The hybrid recommendation system did not apply the metaheuristic strategies to enhance the execution metrics, and the convolution was not minimized in the model to utilize the progressive operators^7^.
- The model did not focus attention on additional learners by characteristics evaluation and updated technologies that were not associated with attaining a better performance in the research^13^.
- PCRS struggled with dealing with new users or courses without sufficient historical data. As the number of users and courses grows, maintaining personalized recommendations becomes computationally expensive^18^.
- The unsupervised nature of RL-MDP means that it does not take task-specific information into account during the transformation process, which could limit its effectiveness in certain scenarios^8^.

### Deployment challenges and potential solutions

The previous methodologies designed for student course recommendation systems encountered several challenges, including difficulty in finding intricate relationships between the course recommendation settings, lack of historical data, computational complexity, lack of larger datasets, lack of generalizability to diverse-aged learners, as well as restricted use in limited resource settings. Hence, to address these challenges, the research intends to develop a Deep Certifier-DX509 model, which includes MA-DLSTM that effectually assesses the sequential data of students and categorizes the essential details within the data that proactively updating course recommendations to match a user’s growing expertise and emerging areas of interest. Further, the integration of the X509 certificate generation algorithm relies on the Proof-of-Work (PoW) consensus algorithm to guarantee the security of blockchain storage. Moreover, the Pow and X.509 certificate algorithms are used in the certificate subsystem to protect the blockchain from unnecessary changes in the research.

## Course recommendation and certificate issue system with deep Certifier-DX509

**Fig. 1:**
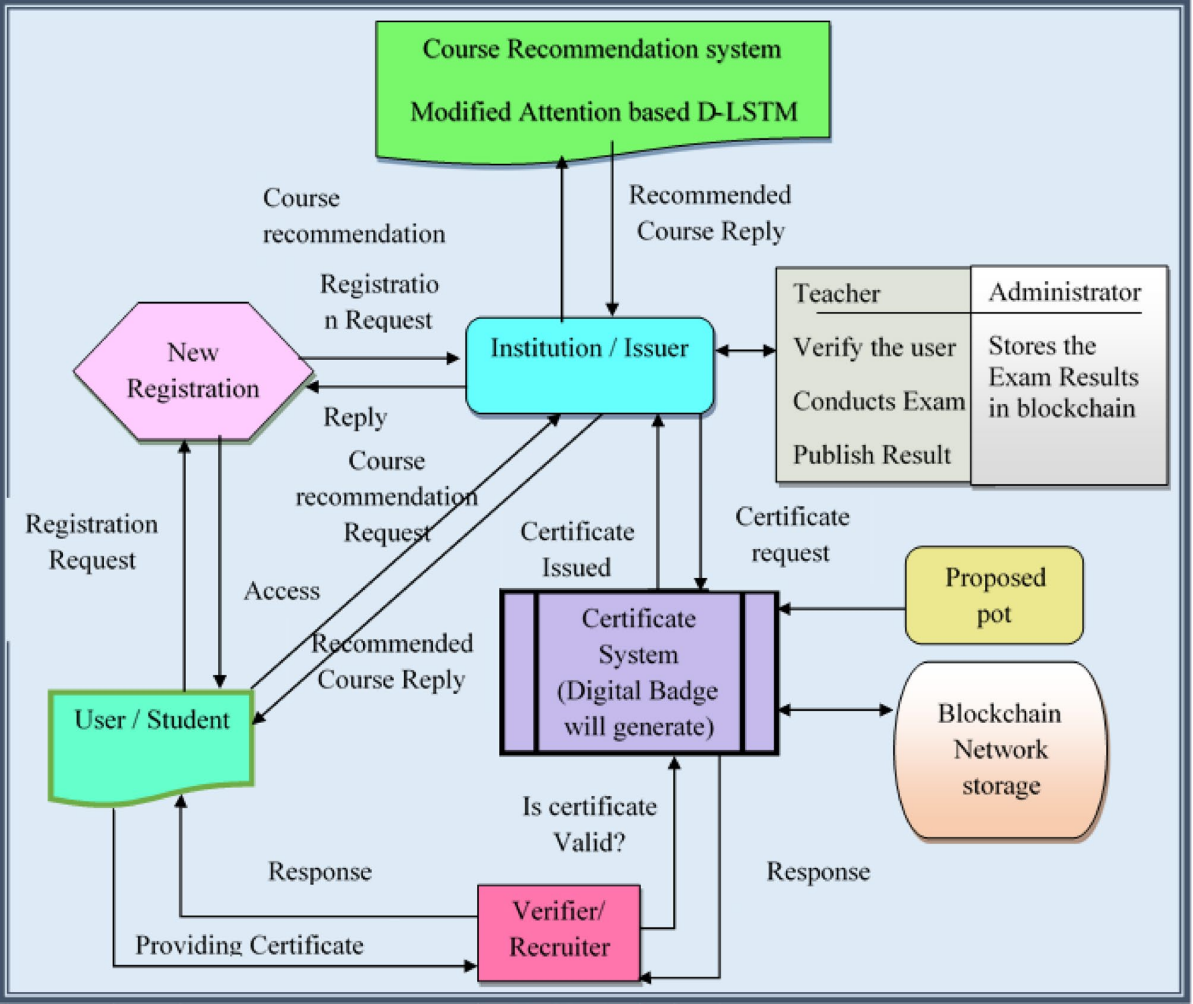
Block diagram of Deep Certifier-DX509 model.

The research majorly concentrates on developing the course recommendation as well as the certificate issuance system with the Deep certifier-DX509. The model works in four phases, where the highly authenticated certificate is issued to the user with high knowledge in the area of study. Initially, the user sends the request to the institution to register the profile, which in turn accepts the request and registers the user with the newly generated username and password, which acts as the access of user. After the registration, the user logs in and requests the institution for the appropriate course to move forward. The course for the user is recommended through the MA-DLSTM model that incorporates the deep LSTM and the modified attention mechanism, which ensures the most significant course recommendation to the user. The model utilizes the course recommendation model dataset^26^ to train the model. The modified attention mechanism in the research is the combination of spatial and positional attention that extracts the spatial and positional features of the input data aggregated from the user. The user with the recommended course is allocated to the teacher, who teaches and examines the performance of the user. Once the examination is conducted, the results are generated with the help of the students’ performance dataset^27^, and the outcomes are stored in the blockchain by the administrator. In case of successful course completion, the user requests the certificate, which is generated through the certificate system by validating the grades scored by the user on the examination. The certificate system utilizes a proposed PoW that checks the validity and is imposed to share the details with the trust anchor to authenticate. The issued certificates are unique to each user. The provided Deep certifier-DX509 model performed the course recommendation and the generation and distribution of certificates significantly. The workflow of the research is illustrated in Fig. 1 as follows.

### Registration phase

The complete course duration of the student in the e-learning platform starts with the user registration. To register the users or the students into the E-Learning Platform or the institution, certain details are fetched from each user. The number of users in the e-learning platform is represented as,

1 $$K=\left\{ {{K_1},{K_2},{K_3},..,{K_b}...{K_r}} \right\}$$

where $K$ denotes the users, and the total number of users in the platform is denoted as, ${K_r}$, and ${K_b}$indicates the ${b^{th}}$user. Each user registers the required data such as name, age, address, gender, academic data, and so on, which are represented as,

2 $$D\left( {{K_b}} \right)={\left\{ {p,q,s,t,u} \right\}_{{K_b}}}$$

where, $p,q$, represents the name and the age of the user respectively, $s$, denotes the residential address of the user, $t$, indicates the gender, and $u$, represents the academic data of the user at the previous academics, which may be the scores in different subjects at different grades. Once the registration is done, users get the information such as roll number, login credentials, Identity number, and separate blockchain private key and address for individual users, where the roll number is the six-digit user-friendly number to recognize the user, Identity number is the admission number that depends on the number of users admitted to the e-learning platform beforehand.

The obtained academic details from the users are signed and stored in the blockchain. To encrypt the data, the Rivest-Shamir-Adleman (RSA) algorithm is utilized in the research. In general, RSA acts as the asymmetric algorithm utilized specifically for the encryption of data and further supports digital signatures^28^. The algorithm ensures high security and provides insurance of confidentiality to the users. To generate a pair of private and public keys, random prime numbers such as, $k$, and $l$, which is utilized to estimate the modules, termed as, $v$. The modulus is the number of public keys in the cryptosystem that should be more than the binary values of each block^29^. The estimation of modulus is represented as,

3 $$v=k * l$$

Further, the public exponent $e$is selected, which lies between 1 and $\phi \left( v \right)$ that shows the relative prime to $k - 1$, and $l - 1$, which is represented as,

4 $$\phi \left( v \right)=\left( {k - 1} \right) * \left( {l - 1} \right)$$

where $\phi$, is the Euler’s totient, the private exponent $h$, is computed as,

5 $$h={v^{ - 1}}\bmod \left( {\phi \left( v \right)} \right)$$

The above equation represents that the private exponent is the multiplicative inverse of the public exponent with Euler’s totient. Thus, the obtained public and private keys are represented as, $pu{b_k}=\left[ {v,e} \right]$, and $pr{i_k}=\left[ {v,h} \right]$ respectively. Thus, the academic data are signed and stored with the generated keys and are represented as, $sign\left( u \right)$.

### Login phase

Once the system is logged in with the credentials obtained during the registration phase, the user is directed to the home page of the server. In the case of the new user, the page suggests registering before logging. The encrypted message for the first given message is converted into the major suitable scheme.

### Course recommendation with modified attention enabled deep LSTM

**Fig. 2:**
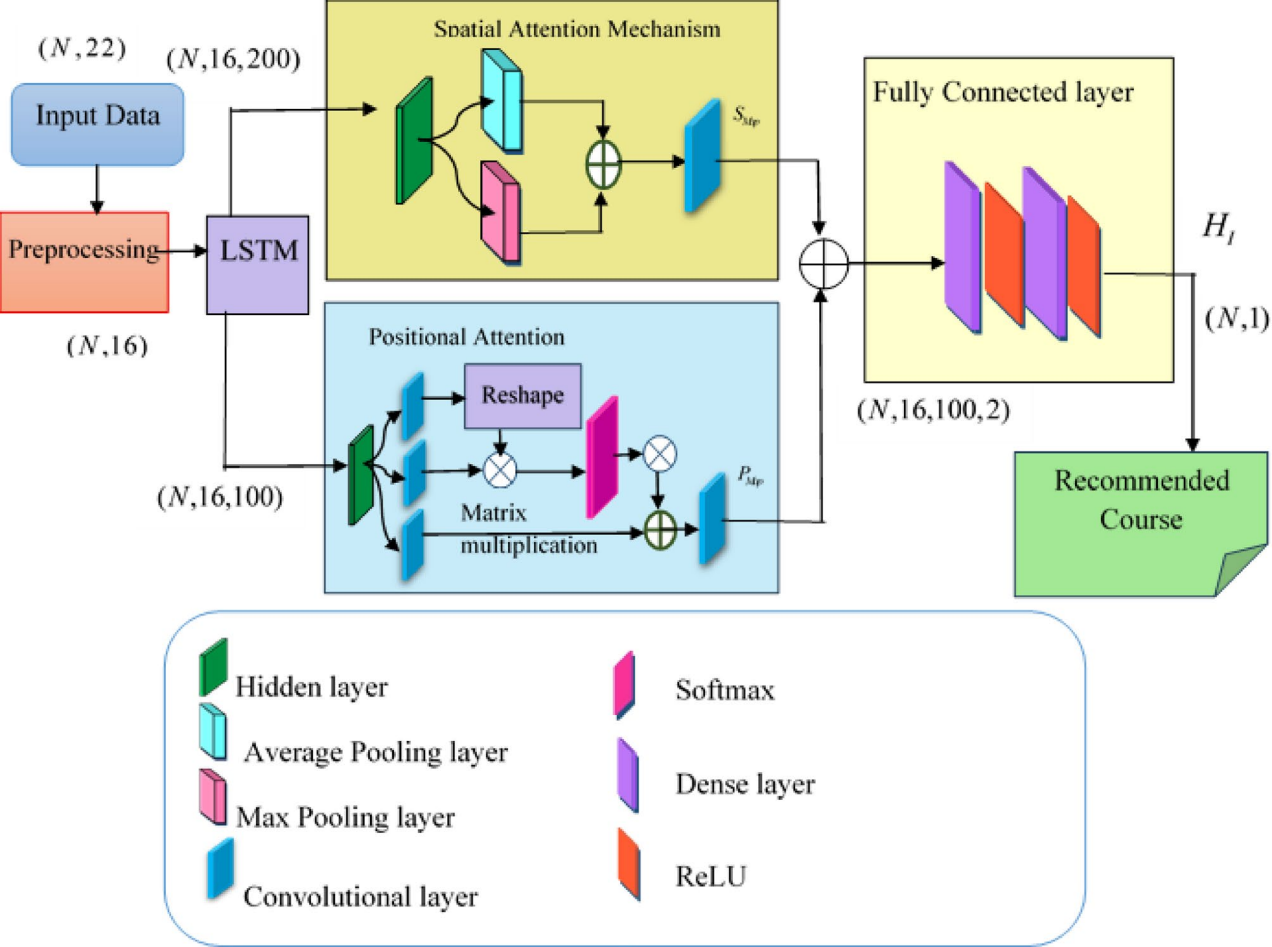
Architecture of the Modified Attention-Enabled Deep LSTM.

Initially, the user interested in learning courses initiates the recommendation advisory from the institution to start the course. The institution, following the prior academic data, tries to suggest the most applicable courses to the user. This recommendation completely depends on the deep learning mechanisms that analyze and suggest accurately. To perform efficient course recommendation, a modified attention-based Deep LSTM is preferred in the research. The model is the combination of the spatial and the positional attention mechanism arranged in parallel to estimate the features, along with the integration of Deep LSTM to recommend the most appropriate courses to the user. The LSTM model extracts intricate patterns and long-range relationships from a user’s course enrollment sequence, furnishing insights into their learning trajectory. Yet, the previous LSTM networks were susceptible to overfitting as well as complexity in capturing difficult affiliations between courses, the computational cost was high, and interpretation complexities. Therefore, such constraints are addressed by incorporating the use of modified attention mechanisms with the deep LSTM networks, as it assists in optimizing the accuracy of personalized recommendations and dynamically adjusting the relevance of past learning experiences based on the present learning situation, also it preserves contextual information across extended time frames in sequential data^23^. The proposed course recommendation model, named MA-DLSTM, is the combination of spatial and positional attention in the deep LSTM model. The proposed model for course recommendation is illustrated in Fig. 2 as follows,

Initially, the input data $(N,22)$ is fetched from the course recommendation dataset^26^ is applied to the preprocessing phase, which selects the required data utilized for the recommendation system. The preprocessed data $(N,16)$ is fed into the LSTM models that learn, process, and classify the sequential data. LSTM is an advanced mechanism of the Recurrent Neural Network (RNN) that overcomes the issues with the capabilities of long-term dependencies. The architecture of the LSTM follows the chain structure having tangent as the activation function. The cell state is the key idea of the LSTM, which acts as the conveyor to transfer and carry linear interactions among the states of the LSTM. Initially, the LSTM decides the information that needs to be the part through the implementation of the sigmoid function, which is otherwise called the layer of a door of oblivion^30^. While using the sigmoid function, the information is discriminated and is represented as,

6 $$f{r_i}=\sigma \left( {{W_{fr}}.\left[ {hi{d_{i - 1}},D\left( {{K_b}} \right)} \right]+{B_{fr}}} \right)$$

where$f{r_i}$ represents forget gate, ${W_{fr}}$ indicates the weights of the forget gate, $hid$ represents the hidden state, ${B_{fr}}$ indicates the bias of the forget gate, and $\sigma$ denotes the sigmoid function. Further, the obtained information is passed into the succeeding layer, which is again processed with the sigmoid function and the tangent function that enables the new candidate, and the cell state is represented^31^ as,

7 $$I{n_i}=\sigma \left( {{W_{In}}.\left[ {hi{d_{i - 1}},D\left( {{K_b}} \right)} \right]+{B_{In}}} \right)$$

8 $$C{l_i}=\sigma \left( {{W_{Cl}}.\left[ {hi{d_{i - 1}},D\left( {{K_b}} \right)} \right]+{B_{Cl}}} \right)$$

where$In$ indicates the input layer with the weights and biases ${W_{In}}$, ${B_{In}}$respectively. The cell state is represented by $Cl$having weights and ${B_{Cl}}$ biases. At last, the final vector is filtered with the sigmoid and the tangent hyperbolic function, where the output is represented as,

9 $$O{t_i}=\sigma \left( {{W_{Ot}}.\left[ {hi{d_{i - 1}},D\left( {{K_b}} \right)} \right]+{B_{Ot}}} \right)$$

where $Ot$ indicates the output layer, with the hidden layer indicated as follows,

10 $$hi{d_i}=O{t_i} \odot \tanh \left( {C{l_i}} \right)$$

The output of LSTM$Ot$ with dimension $(N,16,200)$ is fed into the spatial and positional attention mechanisms that extract the most significant features, replicating the spatial and positional information of the input data. Spatial Attention in the model aids in providing priority in the processing of the data by selectively retrieving the information. The spatial attention map is generated with the features that occur at the inter-spatial relationship in the input, and further, the significance of spatial attention lies in extracting the highly informative part. The average pooling and max pooling operations are applied at the channel axis of the input from the LSTM, which is considered to be more effective in highlighting the most redundant features^14^. Once the max pooling and the average pooling are concatenated, the convolution layer is applied to generate the spatial attention map $(N,16,100)$^32^, which is represented as,

11 $${S_{Map}}=\sigma \left( {{c^{7 \times 7}}\left( {\left[ {Avg\left( {Ot} \right);Maxpool\left( {Ot} \right)} \right]} \right)} \right)$$

Where, $Ot$, is the output from the LSTM, $c$, represents the convolution function with the filter size of $7 \times 7$, and $\sigma$, indicates the sigmoid function. The positional attention initiates the convolution of the obtained features to apply the nonlinear transformations that generate the feature maps $M$, $N$, which are reshaped and then perform a matrix multiplication among the transpose of $M$, and $N$, further, apply the softmax function to estimate the position attention, represented as,

12 $${P_{Map}}=\frac{{\exp \left( {M.N} \right)}}{{\sum {\exp \left( {M.N} \right)} }}$$

Through the same way of execution, the third coefficient is achieved and reshaped to result in the final positional attention map^33^. The obtained map could result in information loss, which is resolved with the multiplication of$\alpha$, the learnable scale parameter, and performing the element-wise multiplication resulted,

13 $$P_{{Map}}^{*}=\alpha \sum {\left( {{P_{Map}}O} \right)} +O{t_{M,N,O,i}}$$

where $O$is the coefficient of the input features. The obtained spatial and positional attention maps are concatenated and are represented as, $T$eith the dimension of $(N,16,100,2)$. The concatenated attention map enables features to be fed into the fully connected module with dense layers, which generates accurate course selection of data with dimension of $(N,16,200)$followed by the ReLU function $(N,50)$, which enhances the course recommendation system. The output is the recommended course for the users represented,${H_I}$have a dimension of$(N,1)$.

### Learning management system

The learning management system involves the teacher and administrator of the institution, along with the user. Once the user is enrolled in the recommended course through the institution with MA-DLSTM, the institution $I$ stores the information of the user ${K_b}$, along with the information of the recommended course, which is depicted as,

14 $$I\left( {{K_b}} \right)=\left\{ {{p_I},{q_I},{t_I},{s_I},{V_I},{H_I},sign\left( {{u_I}} \right),Ad\left( {{K_b}} \right)} \right\}$$

where, $p,q,s,t$, is the information gathered from the user ${K_b}$, at the time of registration, whereas, ${V_I}$, indicates the roll number allocated by the Institution, ${H_I}$, denotes the recommended course to the user, the signature generated for the represented user about their prior academic data is denoted by $sign\left( u \right)$, and $Ad\left( {{K_b}} \right)$, represents the address of the blockchain storage of the user ${K_b}$. With the described information, the institution allocates the teacher to the user. The teacher ensures the involvement of the user or the student and examines to evaluates the participation in the learning process. The examination is conducted, where the appropriate user is authenticated through the Roll Number verification, which is represented as,

15 $${V_I}=V$$

Where, ${V}$, is the given roll number of the user during the login process. Once verified, the examination is completed, and the results are forwarded to the administrator, who ensures and stores the results in the blockchain storage of the institution. In the research, the student’s performance dataset is included through which the results are randomly allocated to all users, and the grades are generated, where the users with a passing grade are provided with the certificate generated in the certificate sub-system. After the successful completion of the course, the user requests the institution for the certificate or badge of their course completion, in which the administrator ensures the results and may pass or fail to issue the certificate.

### Certificate Sub-System

The certificate sub-system mainly concentrates on generating certificates. In the research to generate the certificates, X509 is preferred over the standard authentication. The X509 is the public key certificate standard of cryptography. The certificate includes the information of the issuer to identify, the public key, and the information of the signature. The trusted certificate issued by the X509 has a set of certificate standards that are defined by the ITU-T standardization^34^. The specified certificate is encoded with the abstract syntax notation (ASN) that is stored after converting it into Base64. The utilization of X509 in the generation of certificates aids in achieving a highly secure digital certificate. Further, the X509 aids in digital identity verification, resulting in a reliable way of communication. In addition, the X509 certificates are utilized in several web applications are sources that enhance reliability, interpretability, scalability, and so on^35^. The utilization of the X509 certificates in the organization complies with all standards and regulations of the organization, which effectively complies with all privacy requirements. The additional benefit exhibited in utilizing the X509 certificate is flexibility, as the certificates could be customized to add more extensions or information that are required by the organization^36^. In the research, the generated certificates through X509 are stored in the Blockchain network, where the PoW is added as the consensus protocol to ensure proper transactions are held on the blockchain. There are several protocols, such as proof of stake, proof of elapsed state, and so on, among which PoW is considered in the research due to its ability to protect the blockchain from unnecessary changes^37^. In educational settings, PoW security is a fundamental concept of blockchain technology, which significantly bolsters the integrity of academic credentials such as diplomas and certificates by creating a virtually tamper-proof system where any attempt to alter a record would require an immense computational effort, effectively preventing fraudulent modifications or forgeries, thus furnishes higher level of security^38^. The security of PoW works with the principle that no entity should utilize the processing power more than half of the total power consumption, as the entity that utilizes less power could survive the longest chain. Thus, the generated certificates are stored securely^39^. The digital badge about the certificate is shared with the user based on the request from the user. In the process of certificate issuance, the institution performs the two-step authentication process. Initially, the institution verifies the process with the roll number of the user, and further, with the signed academic data$sign\left( u \right)$. The institution verifies the signature shared by the user with the signature available on the blockchain of the user. If

16 $$sign\left( {{u_I}} \right)=sign\left( u \right)$$

Then, the user is issued with the certificate stored in the blockchain. The user shares the digital badge, and the recruiter verifies it with the issuing authority. The recruiter directly approaches the blockchain storage and ensures the certificate is valid or not. In the case of a valid certificate, the user responds positively. Hence, the integration of the X.509 certificate generator with the PoW security boosts the performance of the research model in improving reliability as well as trustworthiness among learners, as the X.509 certificate generator prevents fraud by establishing a secure digital identity verification process, which effectively restricts access and interactions to unauthorized users. Consequently, the PoW protects against fraudulent recommendations by setting a high barrier to entry, requiring users to prove their identity and computational ability before submitting suggestions, particularly, making it intricate to alter previous transactions, thus improving performance, the Pseudocode for the Course Recommendation and Certificate Issue System is given in Algorithm 1 as follows.

**Algorithm 1:**
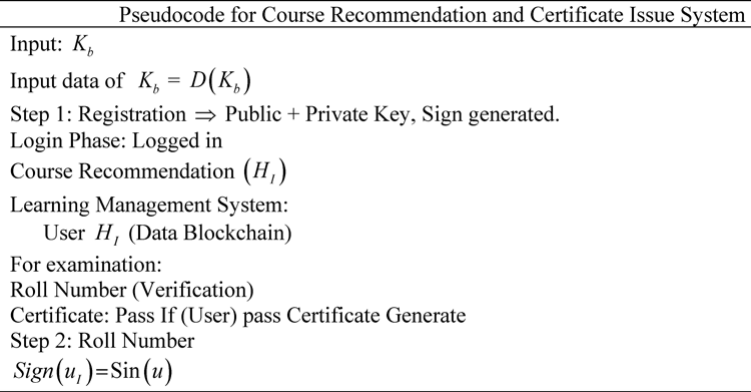
The Deep Certifier-DX509 model.

## Results

The experimental outcomes of the Deep Certifier-DX509 research model are evaluated and analyzed in this section to show the efficiency of the model.

### Experimental setup

The research Deep Certifier-DX509 model is implemented on the PyCharm software equipped with the Python tool running on the Windows 11 operating system utilizing 16 GB of RAM storage and 100 GB ROM. The initial configurations of the proposed network involve Batch size of 32, Activation Function “Linear”, Loss Function “MSE”, default Optimizer Adam, and the learning rate of 0.01.

### Dataset description

**Course recommendation dataset**^26^: This dataset recommends courses to students based on the Skills and Difficulty Level entered by the student. The dataset consists of 3522 rows and 7 columns. The seven columns include Course Name, University, Difficulty Level, Course Rating, Course URL, Course Description, and Skills.

**Students’ performance dataset**^27^: This dataset is a collection of student achievement in secondary education of two Portuguese schools. The dataset consists of 32 attribute columns of CSV files, and attributes include the student grades, demographics, social and school-related features, which were collected by using school reports and questionnaires. The academic performance, including the performance of students in the Maths Subject and the Portuguese language. The dataset has individual attributes such as name, school, age, gender, address, family size, nationality, parents’ education, family size, cohabitation status of parents, and parents’ jobs, etc.

### Performance metrics

The performance of the research is evaluated with the correlation coefficient, Mean Squared Error (MSE), Mean Absolute Error (MAE), and Root Mean Squared Error (RMSE). The evaluation with the metrics showed that the Deep Certifier-DX509 is efficient with better course recommendations and certificate issuance. The obtained values of the metrics are low due to the error metrics that represent the high proficiency outcomes.

### Performance analysis

**Fig. 3:**
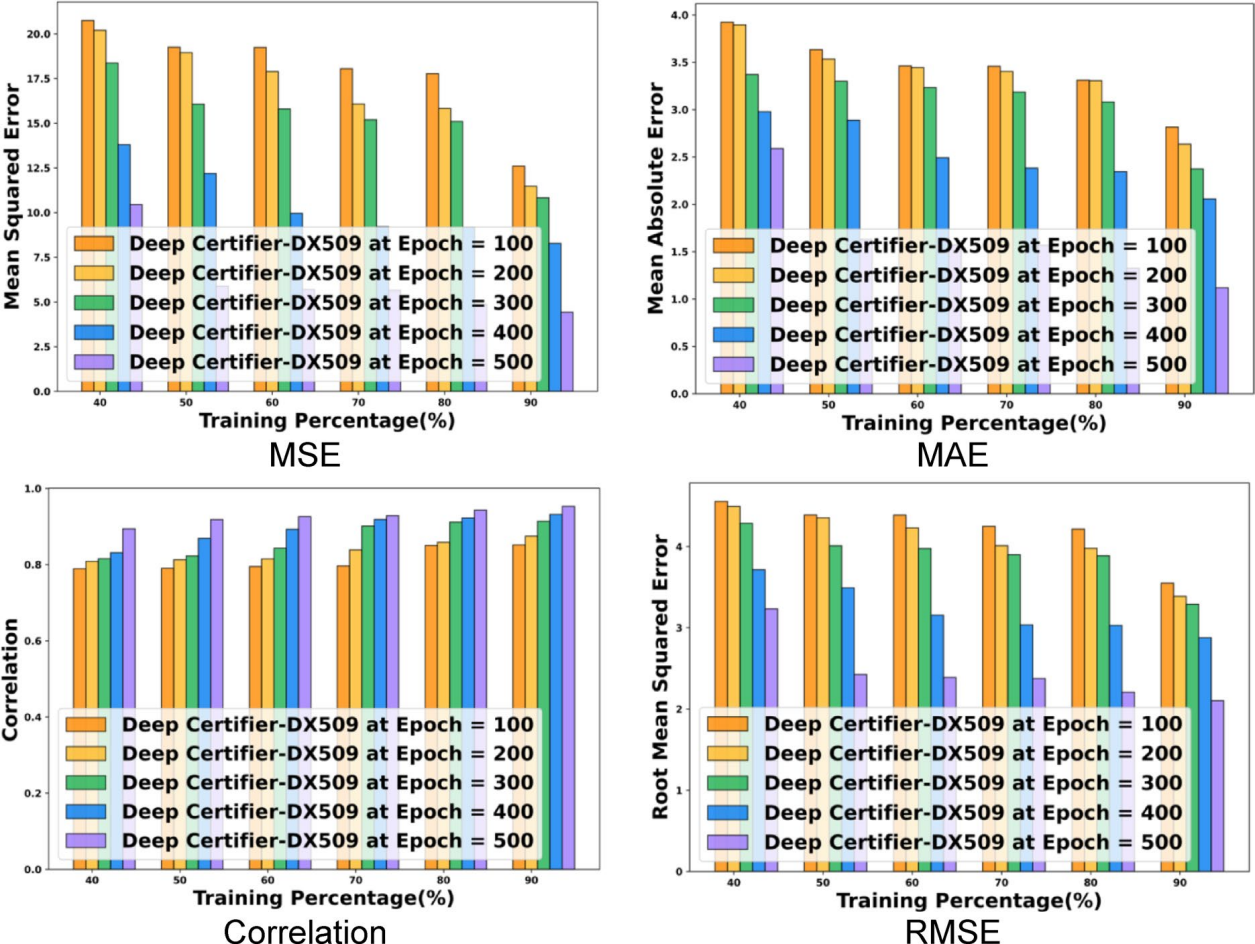
Performance Evaluation of Deep Certifier-DX509.

The performance of the research model Deep Certifier-DX509 is evaluated and achieved 0.952 in terms of correlation. Further, concerning the MSE, the Deep Certifier-DX509 obtains 4.423, which is much less than the other values of TP 90 and epoch 500. The obtained MAE of the model at TP 90 and epoch 500 is 1.119, whereas the RMSE of the given model is 2.1033. The obtained values represent that the model achieved better accuracy in the recommendation with minimal error, which is due to the development of the modified attention-enabled Deep LSTM. The outcomes of the performance evaluation are shown in Fig. 3.

### Comparative analysis

The comparative evaluation of the Deep Certifier-DX509 is carried out with certain existing methods such as RL-MDP^8^, TP-GNN^9^, Hybrid DNN^19^, ELRA^17^, PCRS^18^, KT-Transformers model^22^, LSTM + attention mechanism^23^, Cross-Institutional Blockchain Enrollment System (BCHEEN)^40^, and Elliptic Curve Digital Signature Algorithm (ECDSA)^41^. The proposed model is evaluated using training percentages (TP) from 40, 50, 60, 70, 80, and 90, as well as the K-Fold cross-validation test, 6, 8, and 10. Moreover, the comparative performance outcomes of the research model is detailed in the subsequent sections.

#### Comparative evaluation with training percentage

**Fig. 4:**
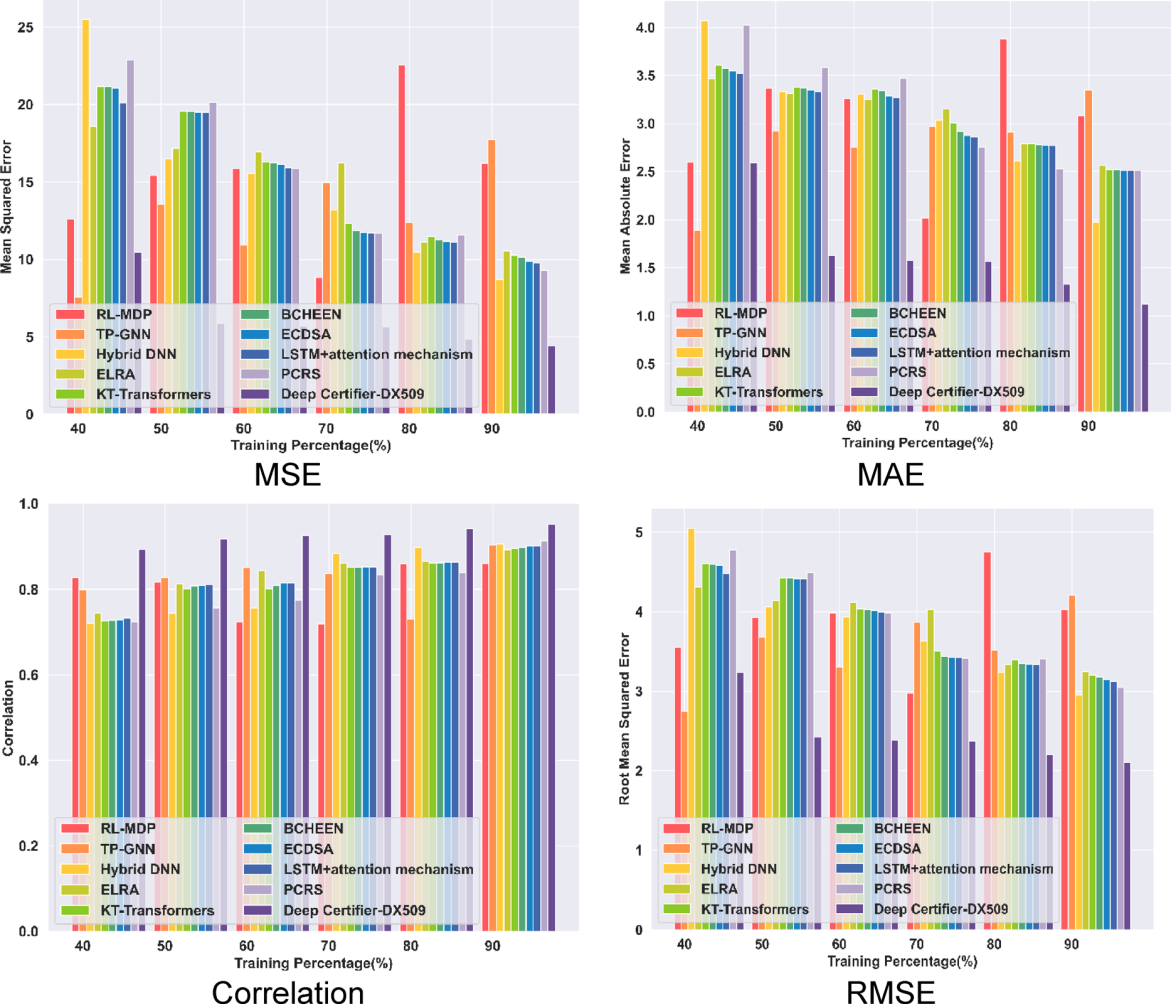
Comparative evaluation of deep certifier-DX509 with training percentage.

The Deep certifier-DX509 model is evaluated and with other existing models based on training percentage, which is illustrated in Fig. 4. The proposed model attained a correlation of 0.95, which is higher compared with the existing methods showed a correlation of 9.68% with RL-MDP and 4.85% with hybrid DNN. Additionally, lesser correlation with the KT-Transformers model by 6.05% and LSTM + attention mechanism by 5.37% in TP 90. Similarly, the proposed Deep certifier-DX509 approach attained minimum MSE compared with the conventional methods, which is an error difference of 11.78 with RL-MDP, 13.31 error difference with TP-GNN method, 6.11 with ELRA framework, and 4.87 with the PCRS model, respectively. Further, the proposed model gained minimum RMSE metrics of 2.18 with TP-GNN, 1.14 error difference with PCRS, and 0.84 with ELRA. The Deep Certifier-DX509 model achieved less MAE of 1.12, compared with others gained a 1.96 error difference with the RL-MDP model, and 2.23 with the TP-GNN model.

#### Comparative evaluation with K-Fold value

**Fig. 5:**
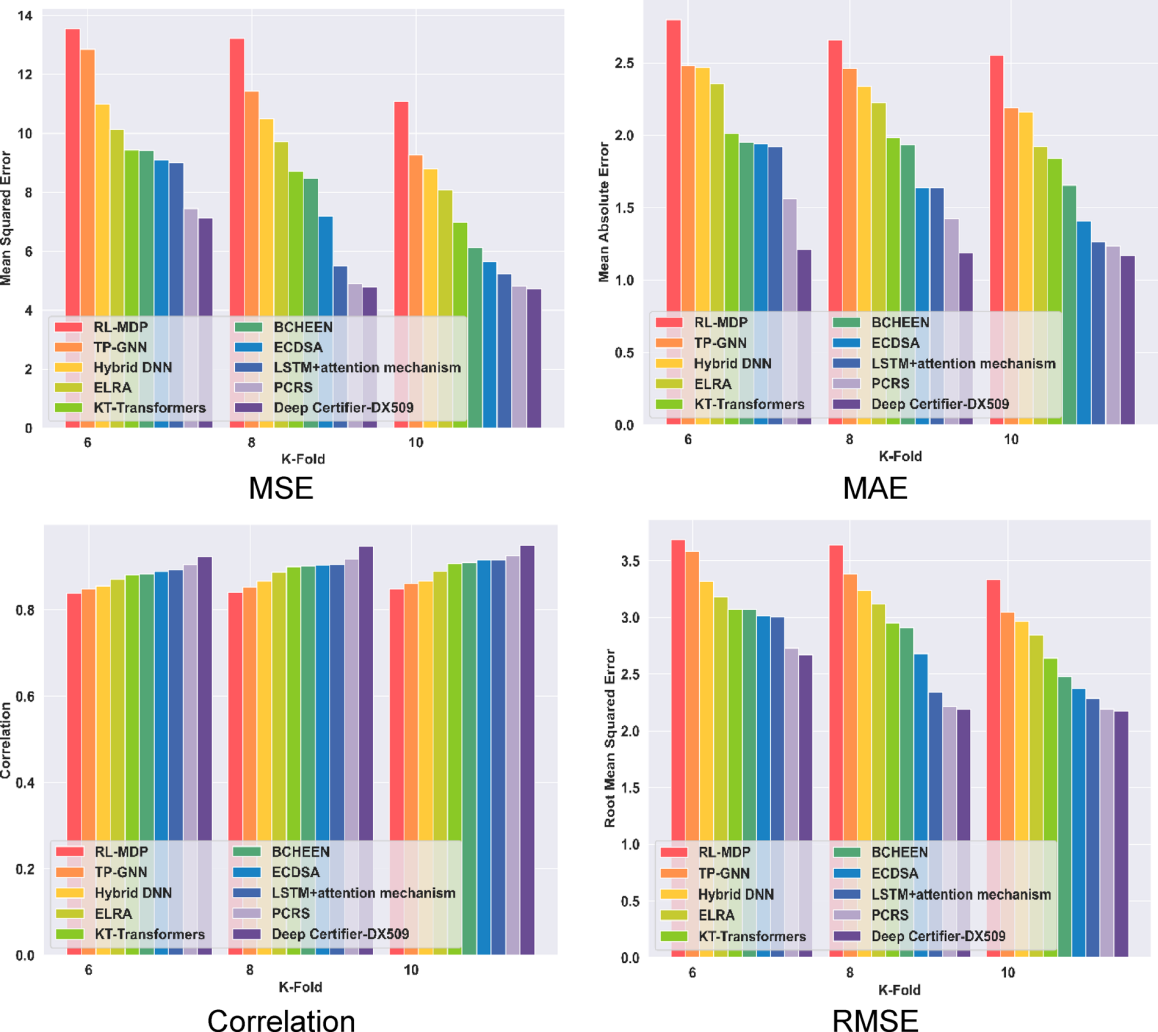
Comparative analysis with K-Fold.

In analysis with correlation, the proposed Deep Certifier-DX509 model attains a 0.95 high correlation for K-Fold 10, while the existing ELRA, as well as TP-GNN methods, acquired 0.89 and 0.86 correlations. In terms of MSE, the proposed Deep Certifier-DX509 model had an MSE of 4.73, which is less compared to the RL-MDP and KT-Transformers models, which have an error difference of 11.09 and 6.98 MSE. In terms of MAE, the proposed model acquired a lower MAE of 1.16, while the existing LSTM + Attention and TP-GNN model has error differences of 1.27 and 2.19. Similarly, the proposed model attained the minimum RMSE of 2.17, which is lower than the BCHEEN and ECDSA models. However, the proposed Deep Certifier-DX509 model achieved low error and demonstrated better performance. The comparative evaluation of the models is shown in Fig. 5.

### Security analysis

The improved security system of the Deep Certifier-DX509 model is evaluated with metrics such as Genuine User Rate, Memory Usage, Responsiveness, throughput, and Transaction Time. The Genuine User Rate is estimated as the number of users who are authorized among the total number of users. The Genuine User Rate is estimated as,

17 $$GUR=\frac{{Aut{h_{users}}}}{{Tota{l_{users}}}},$$

**Fig. 6:**
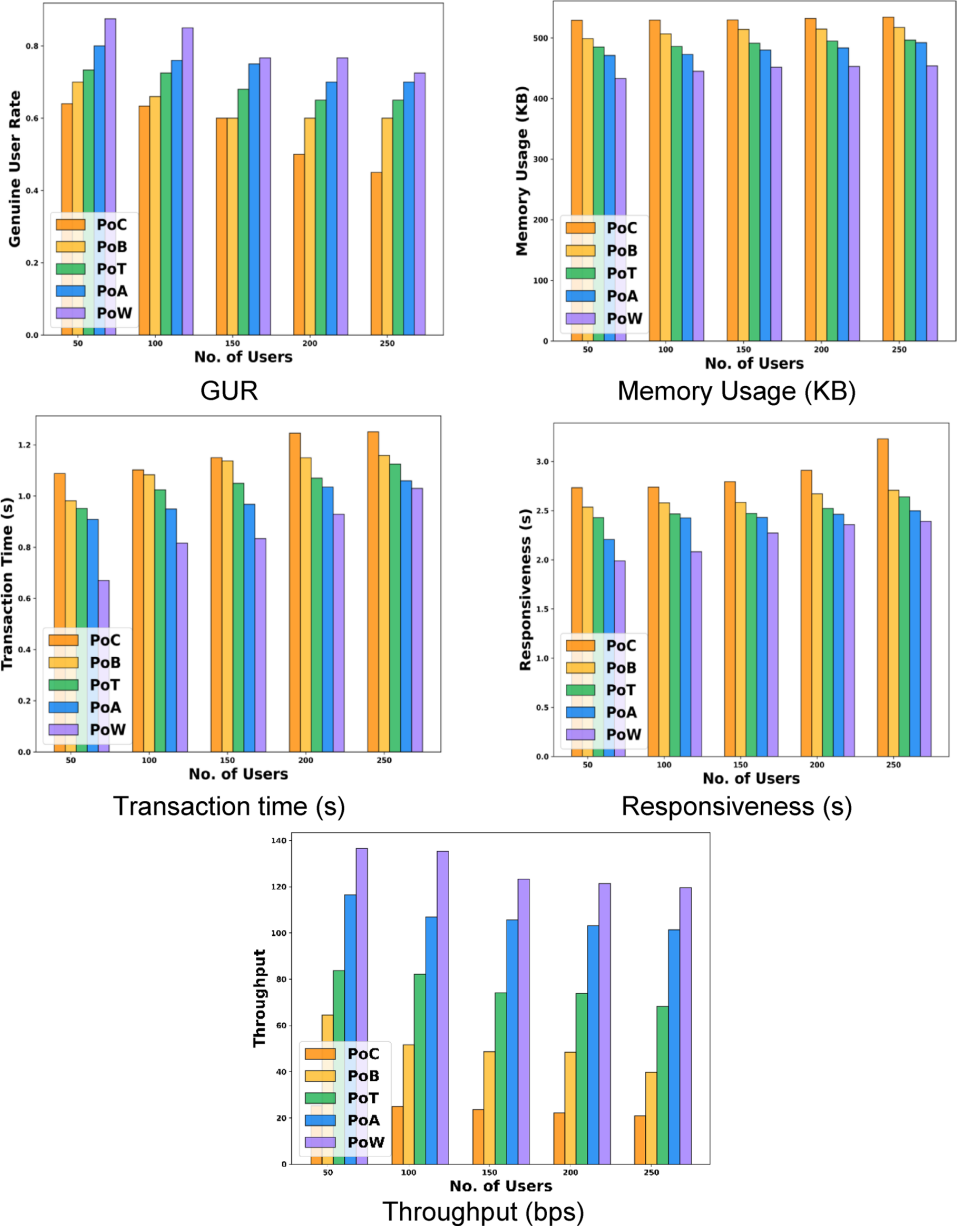
Security Analysis of Deep Certifier-DX509.

Where, $Aut{h_{users}}$, is represented as authorized users and $Tota{l_{users}}$, is indicated as the total users. Memory usage is evaluated as the total memory used for the process, which is estimated as the difference between the energy consumed from the start and the end of the process. The transaction time is represented as the time taken to complete the authentication process. The security analysis of the Deep Certifier-DX509 is compared with different approaches used for verifying transactions and agreement in the blockchain such as, Proof of Capacity (PoC), Proof of Burn (PoB), Proof of Time (PoT), Proof of Authority (PoA), and Proof of Work (PoW) for four different sets of users. While comparing the proposed model with the security analysis techniques, the PoW consumes much less memory usage, and the transaction time consumed by the PoW to complete the authentication process is also much less when compared to the other techniques. Thus, the developed model with the PoW technique achieved high security, and the entire system is authenticated with two-step authentication. Further, the security outcomes of the proposed model in terms of GUR, Memory Usage, Transaction Time, Responsiveness, and Throughput are 0.88, 433.13 KB, 0.67s, 1.99s, and 136bps for 50 users, respectively. In the research, the process is evaluated for GUR and achieved 0.725 at 250 users for the PoW. Further, for several users such as 50, 100, 150, 200, and 250, the GUR achieved 0.88%, 0.85%, 0.77%, 0.77%, and 0.73%. In addition, the memory usage of the users ranges from 50 to 250 users at PoW 433.13 KB, 444.88 KB, 451.66 KB, 452.93 KB, and 453.81 KB, respectively. The transaction time taken to process the single transaction is 0.67s, 0.82s, 0.83s, 0.93s, and 1.03s for the users from 50 to 250. The obtained responsiveness is 1.99s, 2.08s, 2.27s, 2.36s, and 2.39s for 50 to 250 users. Also, the acquired throughput for users from 50 to 250 is 136.65bps, 135.36bps, 123.29bps, 121.28bps, and 119.52bps, respectively. The outcomes are illustrated in Fig. 6. The analysis on security analysis of Deep Certifier-DX509 is shown in Table 2.

**Table 2:** Security Analysis.

Analysis/ Methods		PoC	PoB	PoT	PoA	PoW
For 50 users	GUR	0.64	0.70	0.73	0.80	0.88
	Memory Usage (KB)	529.13	498.85	484.98	471.15	433.13
	Transaction time (s)	1.09	0.98	0.95	0.91	0.67
	Responsiveness (s)	2.73	2.54	2.43	2.21	1.99
	Throughput (bps)	25.23	64.59	83.83	116.39	136.65
For 100 users	GUR	0.63	0.66	0.73	0.76	0.85
	Memory Usage ( KB )	529.43	506.80	485.90	472.72	444.88
	Transaction time (s)	1.10	1.08	1.02	0.95	0.82
	Responsiveness (s)	2.74	2.58	2.47	2.43	2.08
	Throughput ( bps )	24.99	51.66	82.16	106.82	135.36
For 150 users	GUR	0.60	0.60	0.68	0.75	0.77
	Memory Usage ( KB )	529.64	514.13	491.27	479.83	451.66
	Transaction time (s)	1.15	1.14	1.05	0.97	0.83
	Responsiveness (s)	2.79	2.58	2.47	2.43	2.27
	Throughput ( bps )	23.57	48.60	74.15	105.58	123.29
For 200 users	GUR	0.50	0.60	0.65	0.70	0.77
	Memory Usage ( KB )	532.04	514.66	494.84	483.50	452.93
	Transaction time (s)	1.25	1.15	1.07	1.03	0.93
	Responsiveness (s)	2.91	2.67	2.52	2.46	2.36
	Throughput ( bps )	22.08	48.57	73.86	103.14	121.28
For 250 users	GUR	0.45	0.60	0.65	0.70	0.73
	Memory Usage ( KB )	534.24	517.30	496.42	492.18	453.81
	Transaction time (s)	1.25	1.16	1.12	1.06	1.03
	Responsiveness (s)	3.23	2.71	2.64	2.50	2.39
	Throughput ( bps )	20.83	39.78	68.17	101.44	119.52

### Comparative discussion

The conventional methods utilized in the research are RL-MDP^8^, TP-GNN^9^, Hybrid DNN^19^, ELRA^17^, PCRS^18^, KT-Transformers model^22^, LSTM + attention mechanism^23^, Cross-Institutional Blockchain Enrollment System (BCHEEN)^40^, and Elliptic Curve Digital Signature Algorithm (ECDSA)^41^. The conventional methods possessed certain drawbacks, such as RL-MDP relied on the assumption that transforming the action space would lead to better model learning. However, this assumption might not always hold for all environments. Further, the unsupervised nature of RL-MDP means that it does not take task-specific information into account during the transformation process, which could limit its effectiveness in certain scenarios. The design of an effective hybrid architecture required careful tuning and experimentation. Selecting the right combination of neural network components was challenging. Training hybrid DNNs was computationally expensive due to the increased complexity of the model. TP-GNNs model struggled with long-range temporal dependencies, especially when the graph structure changes dynamically over time. The choice of aggregation functions and message-passing mechanisms in TP-GNNs significantly impacted their performance. ELRA’s performance has heavily relied on the quality of attention regularization. Poorly chosen regularization terms led to suboptimal embeddings. ELRA might struggle with capturing complex relationships between items in the recommendation context. PCRS faced difficulty when dealing with new users or courses without sufficient historical data. As the number of users and courses grows, maintaining personalized recommendations becomes computationally expensive. Moreover, the KT-Transformers model had a drawback of overfitting issue, and the required computational cost was higher, while the LSTM + Attention framework was unsuitable for applications requiring expedited processing with large datasets. To tackle these challenges, the proposed Deep Certifier-DX509 model is utilized in the research. The utilization of the X509 performs the authenticated digital certificate generation algorithm. The incorporation of MA-DLSTM generates an accurate recommendation model based on the academic performance of the prior environments. The proposed Deep Certifier-DX509 model achieved high security, and further, the entire system is authenticated with two-step verification. The combination of the Deep LSTM along with the hybridization of the spatial and the positional attention mechanisms ensures the extraction of all feature types from the research. More specifically, the proposed Deep Certifier-DX509 model attained the most appropriate courses for recommendation. Hence, the entire research successfully provides a course recommendation system along with a highly secure digital certificate issuance system. Table 3 provides a comparative discussion of the Deep Certifier-DX509 with other existing techniques.

**Table 3:** Comparative Discussion.

Analysis/ Methods	Training Percentage-90				K-fold 10			
	Correlation	MAE	MSE	RMSE	Correlation	MAE	MSE	RMSE
RL-MDP^5^	0.86	3.08	16.21	4.03	0.85	2.55	11.09	3.33
TP-GNN^6^	0.90	3.35	17.74	4.21	0.86	2.19	9.26	3.04
Hybrid DNN^8^	0.91	1.97	8.70	2.95	0.87	2.16	8.80	2.97
ELRA^1^	0.89	2.56	10.54	3.25	0.89	1.92	8.09	2.84
PCRS^4^	0.90	2.52	10.27	3.21	0.91	1.84	6.98	2.64
KT-Transformers model^35^	0.90	2.52	10.12	3.18	0.91	1.65	6.13	2.48
LSTM + attention mechanism^36^	0.90	2.51	9.87	3.14	0.92	1.41	5.64	2.37
BCHEEN^40^	0.90	2.51	9.75	3.12	0.92	1.27	5.24	2.29
ECDSA^41^	0.91	2.51	9.29	3.05	0.93	1.24	4.81	2.19
**Proposed Deep Certifier-DX509**	**0.95**	**1.12**	**4.42**	**2.10**	**0.95**	**1.17**	**4.73**	**2.18**

### Computational complexity

**Fig. 7:**
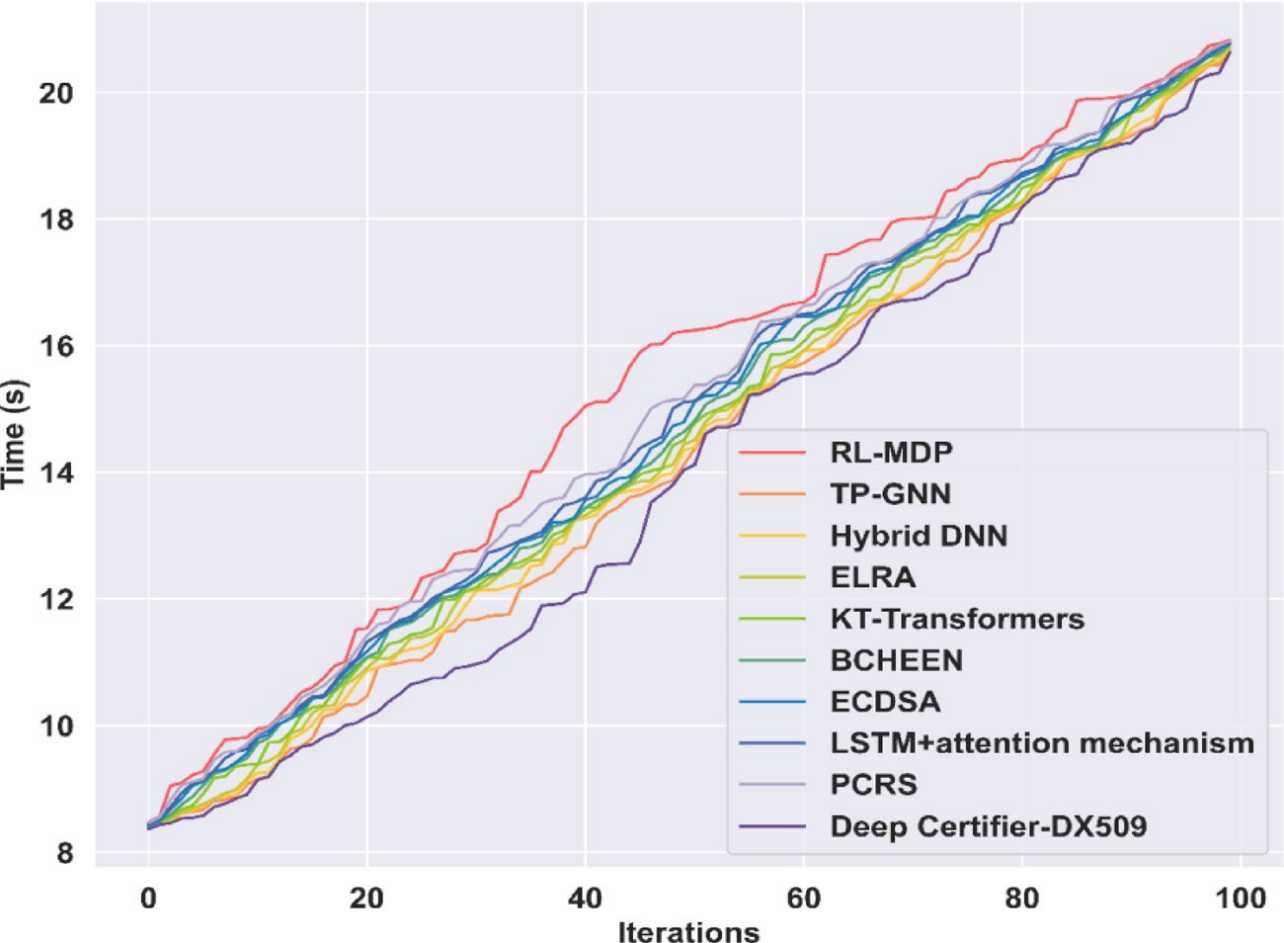
Computational Complexity.

Computational complexity evaluates the computational efficiency of the proposed algorithm over other existing techniques. In this research, the average execution time required for the proposed Deep certifier-DX509 model is 20.64s for epoch 100, which is significantly lower compared to other existing techniques. The overall execution time taken by the existing RL-MDP model is 20.83s, TP-GNN is 20.65s, Hybrid DNN is 20.67s, ELRA is 20.70s, KT-Transformers framework is 20.75s, BCHEEN is 20.76s, ECDSA is 20.77s, LSTM + Attention is 20.78s, and PCRS is 20.81s, which are high compared with the proposed Deep certifier-DX509 framework. Moreover, the proposed approach enables high-speed data processing and improves the computational efficiency of the research model. Figure 7 demonstrates the computational complexity analysis of the proposed model with other existing methods.

## Conclusion

The Deep Certifier-DX509 model is recommended in the research to work with the course recommendation system and with the certificate issuance. The research involves the use of the X509 to perform the authenticated digital certificate generation algorithm with the MA-DLSTM model based on the academic performance of the user in prior environments. Moreover, the conventional approaches utilized for course recommendation system addressed the challenges in capturing the long-range temporal dependencies, inadequate applications in power-constrained systems, high computational complexity, difficulties in providing personalized recommendations for new users, managing contextual suggestions was computationally demanding for the increased number of users or courses, and designing of hybrid architecture required careful tuning and experimentation. These limitations are overcome by the proposed Deep Certifier-DX509 model, which comprises the advantages of Deep learning models like LSTM with the hybridization of the spatial and positional attention mechanisms, which reduce complexity and preserve contextual information across extended time frames in sequential data. Further, the proposed model effectively achieves the most suitable courses for a recommendation system based on personalized contextual analysis, which helps users to choose the courses based on their interests and career goals. Specifically, the utilization of blockchain technology improves the highly secure digital certificate issuance system. The Deep Certifier-DX509 is utilized as the proposed model that ensures the authentication of the digital certificates, as well as the course recommendation models, and outperforms the other existing recommendation models. Moreover, the experimental results illustrate that the proposed model achieved a 0.95 correlation coefficient,1.12 of MAE, 4.42 of MSE, and 2.10 of RMSE based on Training Percentage. Further, the security outcomes of the research model in terms of the GUR, Memory Usage, Transaction Time Responsiveness, and Throughput are 0.73, 453.81 KB, 1.03s, 2.39s, and 119.52bps for 250 users, respectively. Furthermore, the research will focus on developing the model with larger datasets with multiple courses for the application of multiple educational settings in future research.

## Data Availability

Data used in this study are available at: Course recommendation dataset, https://github.com/ShapeLab/ZooidsCompositePhysicalizations/blob/master/Zooid_Vis/bin/data/student-dataset.csv, accessed on April 2024.Student Performance dataset, https://www.kaggle.com/code/ramontanoeiro/student-performance/input, accessed on April 2024.
